# Evaluation of New Diagnostic Biomarkers in Pediatric Sepsis: Matrix Metalloproteinase-9, Tissue Inhibitor of Metalloproteinase-1, Mid-Regional Pro-Atrial Natriuretic Peptide, and Adipocyte Fatty-Acid Binding Protein

**DOI:** 10.1371/journal.pone.0153645

**Published:** 2016-04-18

**Authors:** Mashael F. Alqahtani, Craig M. Smith, Scott L. Weiss, Susan Dawson, Hantamalala Ralay Ranaivo, Mark S. Wainwright

**Affiliations:** 1 Department of Pediatrics, Divisions of Critical Care, Ann & Robert. H. Lurie Children’s Hospital of Chicago, Northwestern Feinberg School of Medicine, Chicago, Illinois, United States of America; 2 Department of Pediatrics, Divisions of Neurology, Ann & Robert. H. Lurie Children’s Hospital of Chicago, Northwestern Feinberg School of Medicine, Chicago, Illinois, United States of America; 3 Ruth D. & Ken M. Davee Pediatric Neurocritical Care Program, Ann & Robert. H. Lurie Children’s Hospital of Chicago, Northwestern Feinberg School of Medicine, Chicago, Illinois, United States of America; 4 Department of Anesthesiology and Critical Care Medicine, The Children’s Hospital of Philadelphia, Perelman School of Medicine at the University of Pennsylvania, Philadelphia, Pennsylvania, United States of America; 5 Department of Pathology and Laboratory Medicine, Swedish Covenant Hospital, Chicago, Illinois, United States of America; Bambino Gesù Children's Hospital, ITALY

## Abstract

Elevated plasma concentrations of matrix metalloproteinase-9 (MMP-9), tissue inhibitor of metalloproteinase-1 (TIMP-1), mid-regional pro-atrial natriuretic peptide (mrProANP), and adipocyte fatty-acid-binding proteins (A-FaBPs) have been investigated as biomarkers for sepsis or detection of acute neurological injuries in adults, but not children. We carried out a single-center, prospective observational study to determine if these measures could serve as biomarkers to identify children with sepsis. A secondary aim was to determine if these biomarkers could identify children with neurologic complications of sepsis. A total of 90 patients ≤ 18 years-old were included in this study. 30 with severe sepsis or septic shock were compared to 30 age-matched febrile and 30 age-matched healthy controls. Serial measurements of each biomarker were obtained, beginning on day 1 of ICU admission. In septic patients, MMP9-/TIMP-1 ratios (Median, IQR, n) were reduced on day 1 (0.024, 0.004–0.174, 13), day 2 (0.020, 0.002–0.109, 10), and day 3 (0.018, 0.003–0.058, 23) compared with febrile (0.705, 0.187–1.778, 22) and healthy (0.7, 0.4–1.2, 29) (*p*< 0.05) controls. A-FaBP and mrProANP (Median, IQR ng/mL, n) were elevated in septic patients compared to control groups on first 2 days after admission to the PICU (*p* <0.05). The area under the curve (AUC) for MMP-9/TIMP-1 ratio, mrProANP, and A-FaBP to distinguish septic patients from healthy controls were 0.96, 0.99, and 0.76, respectively. MMP-9/TIMP-1 ratio was inversely and mrProANP was directly related to PIM-2, PELOD, and ICU and hospital LOS (*p*<0.05). A-FaBP level was associated with PELOD, hospital and ICU length of stay (*p*<0.05). MMP-9/TIMP-1 ratio associated with poor Glasgow Outcome Score (*p*<0.05). A-FaBP levels in septic patients with neurological dysfunction (29.3, 17.2–54.6, 7) were significantly increased compared to septic patients without neurological dysfunction (14.6, 13.3–20.6, 11). MMP-9/TIMP-1 ratios were significantly lower, while A-FaBP and mrProANP were higher in septic patients compared to the control groups. Each biomarker was associated with hospital morbidity and length of stay. These results suggest that these biomarkers merit further prospective study for the early identification of children with sepsis.

## Introduction

Sepsis remains a major cause of morbidity and mortality in pediatrics, with over 75,000 hospital admissions and $4.8 billion in health care costs in the United States annually [1]. The prevalence of pediatric severe sepsis in intensive care units in the United States has increased over the last decade from 6% in 2004 to ~8% in 2012, with a point prevalence of 8% globally [2,3].

The use of biomarkers has the potential to improve early recognition of patients with sepsis. Matrix metalloproteinases (MMPs) are a family of zinc-containing metalloendopeptidases involved in cell remodeling, adhesion, and apoptosis [4]. MMPs are secreted as pro-MMPs, which are activated by a variety of proteinases. MMP activity is highly regulated by interaction with tissue inhibitors of metalloproteinase (TIMPs) and by α-macroglobulins [5]. MMP-9, TIMP-1levels, and MMP-9/TIMP-1 ratio have previously been studied as biomarkers in adult severe sepsis and septic shock [6–8]. Pro-atrial natriuretic peptide (ProANP), the precursor of ANP and primary form of ANP storage in atrial cardiomyocytes, participates in the innate and adaptive immune response. ProANP levels in adults with sepsis are significantly higher in non-survivors than in survivors [9]. Circulating levels of adipocyte fatty acid–binding protein (A-FaBP), an intracellular lipid-binding protein, have been linked to severity of atherosclerosis, cardiovascular disease events, ischemic stroke, and sepsis [10]. None of these biomarkers have been evaluated for identification of sepsis in children.

Studies in adults have also proposed these biomarkers for the detection of neurologic injury. Increases in MMP-9 and TIMP-1 have been associated with compromise of the blood brain barrier (BBB) [11–15]. MrProANP and A-FaBP have been associated with identification of ischemic stroke and prognosis for recovery [16–18].

The objectives of this study were to investigate the utility of MMP9, TIMP1, mrProANP, and A-FaBP as diagnostic biomarkers in pediatric patients with severe sepsis and septic shock. Based on the precedent from adult data, a secondary aim was to investigate their ability to detect concomitant neurological injuries.

## Materials and Methods

The current investigation is a secondary analysis of an existing, previously published cohort [19]. This prospective, observational study was performed in a 42-bed pediatric intensive care unit (PICU) at an academic medical center between May 2009 and December 2010. The prospective study was approved by the Institutional Review Board at Children’s Memorial Hospital (now Ann & Robert H. Lurie Children’s Hospital of Chicago) and written informed consent/assent was obtained from each patient, parent, next of kin or guardian. Details of patient enrollment and data collection are provided in [19]. An amendment to the prospective study to allow use of the samples for analysis in the present study was subsequently also reviewed and approved by the Institutional Review Board at Ann & Robert H. Lurie Children’s Hospital of Chicago.

### Patient population

Study samples were obtained from patients consecutively admitted to the PICU who were ≤ 18 years-old, and met criteria for severe sepsis or septic shock as defined by the International Pediatric Consensus Conference [20] (septic patients). Exclusion criteria were cardiac arrest before admission, treatment with inhaled NO or sildenafil, supplementation with arginine, citrulline, or carnitine, a metabolic, urea cycle, or mitochondrial disorder, chronic renal or hepatic impairment, unrepaired cyanotic heart disease or single-ventricle anatomy, major surgery within the previous 72 hours, transfer from another facility with ongoing sepsis >24 hours, and prior study enrollment. Age-matched control patients from the same hospital were enrolled into two groups; (a) febrile patients (temperature ≥ 38.5°C) evaluated for infection without severe sepsis or shock (febrile controls) and; (b) healthy controls, comprising afebrile patients without evidence of an active infectious or inflammatory condition (e.g. patients undergoing endoscopy for chronic abdominal pain. We did not record data on which organisms were discovered in the febrile control group. Additional details are provided in the previous results from this study group [19].

### Study Measurements

For septic patients, blood was collected within 24 hours of admission (day 1), and then daily for seven days or at PICU discharge (whichever came first) for measurement of biomarkers. If consent could not be obtained within 24 hours of admission, study labs were measured on discarded blood from the earliest clinical samples drawn on day 1. Due to limited availability of discarded blood and the requirement that blood draws be timed with clinical sampling, not all study labs were measured on every day.

Blood specimens were processed upon collection in the main hospital laboratory. MMP-9 and TIMP-1 levels were measured using an enzyme-linked immunosorbent assay (R&D Systems, Minneapolis, MN). Mr-ProANP levels were measured using an automated immunofluorescent assay (ThermoFisher Brahms, Middletown, VA). A-FABP levels were measured using a commercially available ELISA (Biovendor, Asheville, NC).

### Outcome Measures

The primary study outcome was the difference in plasma biomarker concentration between septic patients and febrile and healthy controls. Secondary outcomes included, the association of each biomarker with; (a), length of stay (LOS); Pediatric Index of Mortality (PIM)-2 score; (c) daily Pediatric Logistic Organ Dysfunction (dPELOD) scores, and (d) Glasgow outcome score (GOS) [21] at 6 months following hospital discharge. Only 2 (7%) of the sepsis cohort died and mortality was not included as an outcome measure. Neurologic function was classed as normal or abnormal based on the worst documented clinical exam on each study day using consensus criteria for neurologic dysfunction in sepsis (Glasgow Coma Score ≤ 11 or acute change in mental status with a decrease in GCS ≥ 3 points from abnormal baseline) [20].

### Statistical Analysis

Statistical analysis was performed using Statistical Package for the Social Sciences (SPSS Version 12.1, Chicago, IL) and SAS 9.2 (SAS institute Inc., Cary, NC). Wilcoxon rank-sum or Kruskal-Wallis tests were used to compare continuous and chi-square or Fisher’s exact test for dichotomous variables. Longitudinal changes of repeated measurements in septic patients were analyzed using linear mixed modeling with a random intercept. Associations of biomarkers with organ dysfunction (dPELOD), severity of illness (PIM-2) and clinical outcome (LOS, inotrope-free days, ventilator-free days, and GOS) were tested using Spearman’s coefficient with Dunn’s correction for multiple comparisons. In the sepsis group, A-FaBP and mrProANP levels were measured in pooled serum samples from days 1 and 2 after ICU admission in order to provide sufficient samples for analysis. The area under the receiver operating characteristic curve (AUROC) was used to determine the discrimination of MMP-9/TIMP-1 ratio, A-FaBP and mrProANP levels to differentiate septic children from controls and to identify neurologic dysfunction. P-values ≤0.05 were considered significant.

## Results

There were 1789 patients consecutively admitted to the PICU and 85 (4.8%) met criteria for severe sepsis or septic shock. We excluded 37 patients for not meeting the inclusion criteria; only 30 (63%) patients consented to enrollment of the remaining 48 eligible septic patients. Two control groups of 30 control patients were enrolled: age-matched febrile and age-matched healthy patients. As we have previously reported [19], there were no significant differences between groups in age, gender, or ethnicity. Both PIM-2 and PELOD scores were higher in the sepsis group (all p<0.001).

### MMP-9 and TIMP-1 as clinical biomarkers

**Fig 1:**
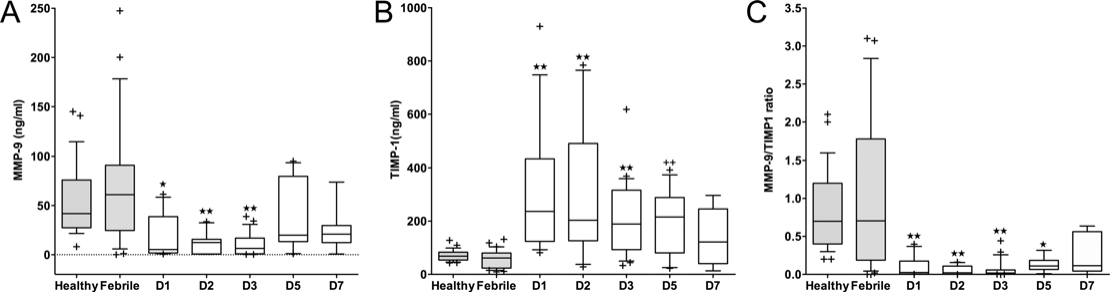
Box and whiskers plots for MMP-9 and TIMP-1 levels, and MMP-9/TIMP-1 ratio in septic patients compared to controls. **(A)** MMP-9, (B) TIMP-1 levels (ng/ml), and (C) MMP-9/TIMP-1 ratios in healthy and febrile controls (gray boxes) compared to septic patients (white boxes) on days-1, 2, 3, 5, and 7 after ICU admission. Data are presented as median, interquartile range, and whiskers indicating 10^th^ to 95^th^%ile (Outliers represented as +). (A) * P <0.05, ** P <0.01 versus healthy and febrile controls. (B) ** P <0.01 versus healthy and febrile controls; *P <0.05 versus febrile controls. (C) ** P <0.01 versus healthy and febrile controls; *P <0.05 versus healthy controls.

MMP-9 levels (Median, IQR ng/mL, n) for the sepsis group on day 1 (5.3, 1.8–38.7, 13), day 2 (12.4, 0.7–15.7, 11), and day 3 (6.4, 0.9–16.9, 22) were lower than febrile (61, 24.7–90.9, 28) and healthy controls (41.8, 27.4–76.0, 29); (*p*< 0.05 for days 1, 2, and 3). MMP-9 levels were not significantly different between groups on days 5 and 7 (Fig 1A). TIMP-1 levels on day 1, 2, and 3 were higher; day 1 (236.8, 124.1–434.2, 13), day 2 (203.2, 126.2–784.3, 11), and day 3 (189.1, 93.1–316.3, 23) compared with febrile (61.6, 23.7–80.5, 30) and healthy (68.3, 53.7–83.3, 29) (*p*< 0.05 for day 1, 2, 3, and 5) (Fig 1B).

A reduction in MMP-9/TIMP-1 ratio (Median, IQR, n) has been reported in adults with sepsis [22]. In the sepsis group this ratio was significantly decreased on days 1, 2, and 3; day 1 (0.02, 0.005–0.2, 13), day 2 (0.02, 0.002–0.1, 10), and day 3 (0.02, 0.004–0.06, 23) compared with febrile (0.7, 0.2–2.0, 22) and healthy (0.7, 0.4–1.2, 29) controls (*p*< 0.05 for day 1, 2, 3,and 5). On day 7, there were no significant intergroup differences for TIMP-1 levels or MMP-9/TIMP-1 ratios.

### A-FaBP and MrProANP as clinical biomarkers of sepsis, and comparison to C-reactive protein

**Fig 2:**
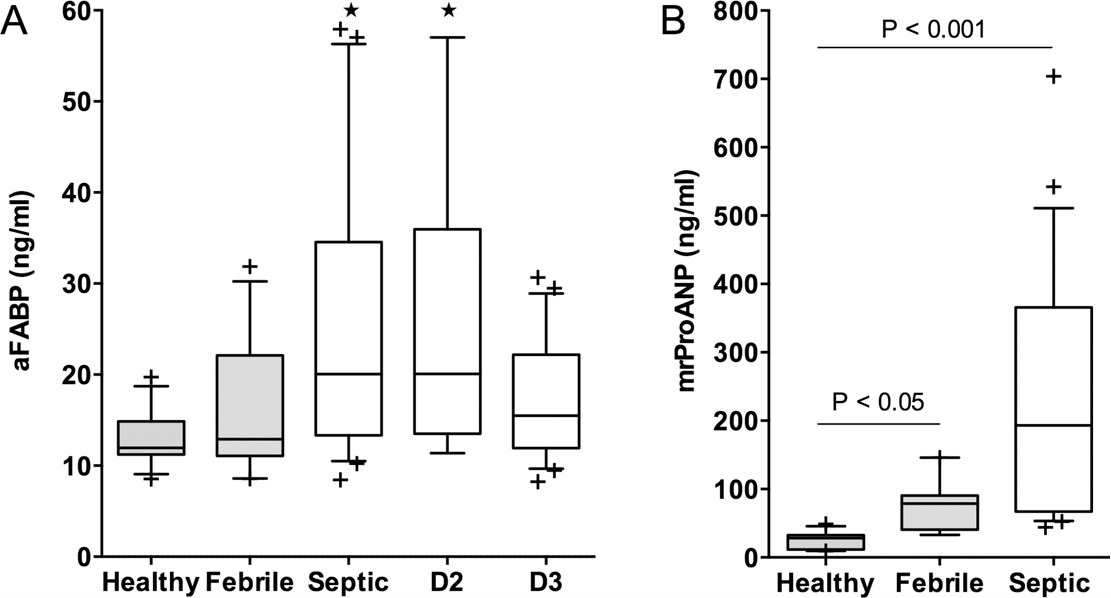
Box and whiskers plots for A-FaBP and mrProANP levels in septic patients compared to controls. (A) A-FaBP levels (ng/ml) in healthy and febrile controls (gray boxes) compared to septic patients on days 1–2 (combined samples) and day 3 of ICU admission (white boxes). (B) mrProANP levels (ng/ml) in healthy and febrile controls (gray boxes) compared to septic patients (pooled samples for days 1–2) (white boxes). Data are presented as median, interquartile range, and whiskers indicating 10^th^ to 95^th^%ile (Outliers represented as +). (A) * P <0.05 versus healthy controls.

In the sepsis group, A-FaBP and mrProANP levels (Median, IQR ng/mL, n) were measured in pooled serum samples from days 1 and 2 after ICU admission. A-FaBP levels for septic patients (20.1, 13.3–34.6, 22) were elevated compared to healthy (11.9, 11.2–14.9, 14;*p*<0.05), but not febrile controls (12.9, 11.0–22.1, 14) *(*Fig 2A). MrProANP levels for septic patients (193.0, 67.2–365.6, 21) were also significantly increased compared to healthy controls (28.3, 11.6–32.511), but not compared to the febrile group (78.7, 40.8–90.4, 9). Levels for febrile controls were significantly increased compared to healthy controls (*p*< 0.05) (Fig 2B).

To compare these measures against an established biomarker we examined the correlation for each with C-reactive protein (CRP) for the sepsis cohort. We did not have sufficent sample to carry out similar analyses for either control group. In the sepsis group, CRP levels measured on day 1 of admission to the ICU were 11.4±2.2 ng/ml (n = 24). There was no significant correlation between Day 1 CRP levels and mrProANP (r^2^, 0.07; p = 0.32), FABP (r^2^, 0.04; p = 0.48) or MMP/TIMP-1 ratio (r^2^, 0.0004; p = 0.93).

### Receiver operating characteristics analyses

**Fig 3:**
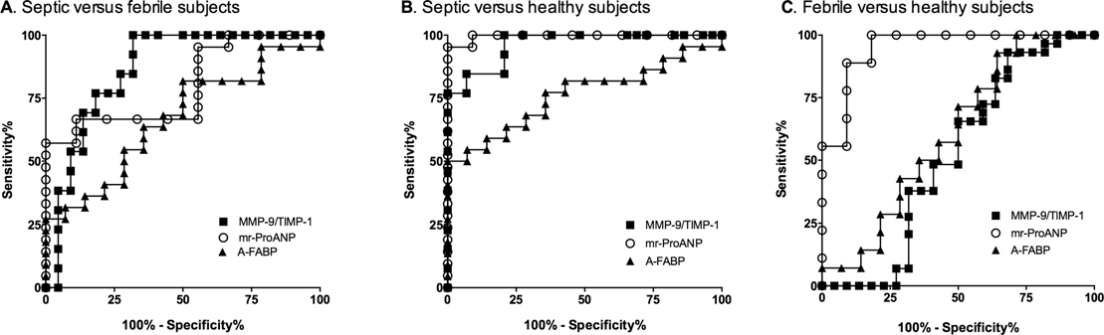
Receiver operating characteristic curves for biomarker discrimination between septic versus control febrile and healthy subjects. ROC curves for MMP-9/TIMP-1 ratio, mr-ProANP and A-FABP levels for (A) septic versus febrile subjects; (B) septic versus healthy subjects and (C) febrile versus healthy subjects. Triangle, A-FABP levels; Circle, mr-ProANP levels; Square, MMP-9/TIMP-1 ratio.

The AUROC for MMP-9/TIMP-1 ratios on day 1 to discriminate septic patients from febrile controls was 0.86, (95% CI 0.74, 0.96), (Fig 3A) and from healthy controls was 0.96, (95% CI 0.91, 1.0 (both *p*<0.001) (Fig 3B). This ratio did not discriminate between febrile and healthy controls (0.52, 95% CI 0.34, 0.70) (Fig 3C). The MMP9/TIMP1 cut-point of < 0.36 yielded a sensitivity of 95% and specificity of 70% to diagnose sepsis.

The AUROC for A-FaBP levels to discriminate between septic and healthy patients was 0.76 (95% CI 0.61, 0.92; *p*<0.05) (Fig 3B). The AUROC for A-FABP to detect a difference between septic and febrile patients (0.67 95% CI 0.49, 0.85) (Fig 3A) or between febrile and healthy controls (0.61 95% CI 0.39, 0.82) were not significant (Fig 3C) was not significant. The A-FaBP cut-point of >13.35 yielded a sensitivity of 77% and specificity of 64% to diagnose sepsis.

The AUROC for mrProANP to detect a difference septic and febrile patients was 0.79 (95% CI 0.63–0.96; *p*<0.05) (Fig 3A) and with healthy patients was 0.99 (95% CI 0.86, 1.0; p<0.001) (Fig 3B). The AUROC for mrProANP to detect a difference between febrile and healthy controls was 0.95 (95% CI 0.86–1.0) (Fig 3C). A a cut-point of > 38.5 yielded a sensitivity of 100% and specificity of 90% to diagnose sepsis.

### Relation of biomarkers to illness severity and 6 month outcome

In septic patients, day 1 plasma MMP-9/TIMP-1 ratio mrProANP levels were related to PIM-2, PELOD, and ICU and hospital LOS (*p*<0.05) (Table 1). A-FaBP level was associated with PELOD, hospital and ICU length of stay (*p*<0.05). MMP-9/TIMP-1 ratio on D1 was associated with GOS (*p*<0.05), but mrProANP and A-FaBP were not (Table 1). We collected daily maximum ionotrope score and classified septic patients as having “rapid recovery” if IS was <5 or “slow recovery” if IS remained >5 on day 3 or later [19]. There was no significant difference in mrProANP levels between the slow (200.8±58.7), and fast (276.7±53.2; p > 0.05) responders.

**Table 1:** Association of study measurements with severity of illness, organ dysfunction, and clinical outcome in septic patients^a^.

		MMP-9/TIMP-1		MrProANP		A-FaBP	
Outcomes		rs	P	rs	P	rs	P
Severity of illness and Organ Dysfunction	PIM-2	-0.57	<0.001	0.60	<0.001	0.25	0.092
	PELOD	-0.74	<0.001	0.62	<0.001	0.36	0.013
Clinical Outcome	ICU LOS	-0.68	<0.001	0.69	<0.001	0.37	0.011
	Hospital LOS	-0.66	<0.001	0.62	<0.001	0.34	0.020
	Inotrope-free days	0.23	0.299	-0.07	0.748	-0.24	0.337
	Ventilator-free days	-0.242	0.277	-0.16	0.497	-0.43	0.072
	GOS^**b**^	0.45	0.036	-0.19	0.404	0.014	0.956

MMP-9, matrix metalloproteinase-9; TIMP-1, tissue inhibitor of metalloproteinase-1 (TIMP-1); mrProANP, mid-regional pro-atrial natriuretic peptide; A-FaBP, adipocyte fatty-acid-binding proteins; PIM-2, Pediatric Index of Mortality-2 score, PELOD, Pediatric Logistic Organ Dysfunction score; LOS, length of stay; GOS, Glasgow outcome score

^a^ Spearman’s rank correlation coefficient (rs) refer to day 1 values

^b^ GOS at 6 months is dichotomized into poor (score of 3–5) or good (score of 1 or 2) outcome

### Relation of biomarkers to neurologic function and complications

Eight (27%) of the sepsis group had a history of neurologic disease including CNS tumors (4), developmental delay, hypoxic-ischemic encephalopathy or chromosomal disorder (Trisomy 21 and VACTERL). Only 1 (3%) of the sepsis group underwent CNS imaging during hospitalization. This patient had confirmed meningitis and the study showed diffuse cerebral edema. We examined the association of each biomarker (data expressed as median, IQR, n) with new onset sepsis-associated neurologic dysfunction [20]. A-FaBP levels in patients with neurological dysfunction (29.3, 17.2–54.6, 7) were significantly increased compared to patients without neurological dysfunction (14.6, 13.3–20.6, 11; p < 0.05 by Mann-Whitney test). AUROC for A-FaBP was (0.81, 95% CI 0.55, 1.06, p = 0.03). MMP-9/TIMP-1 ratio did not differ between patients with (0.12, 0.02–0.20, 7) and those without neurologic dysfunction (0.01, 0.00–0.08, 15). mrProANP levels also showed no significant differences between these 2 groups (220.3, 105.6–365.6, 9 *vs* 127,0, 61.4–358.8, 12). The AUROC for the MMP-9/TIMP-1 ratio (0.76, 95% CI 0.56, 0.97, p = 0.05) and mrProANP levels. (0.57, 95% CI 0.32, 0.83, p = 0.57) to discriminate between these 2 groups was not statistically significant.

## Discussion

The principal findings of this pilot study are the potential for MMP-9/TIMP-1 ratios, A-FaBP and mrProANP levels to serve as biomarkers for the identification of sepsis in pediatric patients. The reduction in the MMP-9/TIMP-1 ratio in septic patients has not previously been reported in children, and our finding is consistent with previous reports in adults [7]. Neither mrProANP nor A-FaBP have previously been studied in children with sepsis, but both show promise based on this single center study. A-FaBP levels discriminated between patients with and those without neurologic dysfunction, consistent with its precedent as a biomarker for stroke in adults. Despite the reported association of mrProANP with neurologic insults or prognosis in adults, we found no such association, although our study was underpowered to test this hypothesis, and was confounded by the inclusion of patients with pre-existing neurologic conditions. Last, a strength of this study is the use of febrile controls in addition to a healthy control group as is often used in studies of biomarkers in sepsis. Previous studies have compared patients with sepsis to healthy controls, but not to febrile controls [6, 8, 22].

The role of MMP-9 role in sepsis may be related to its function in the cleavage of collagen in the basement membrane, thereby enabling leukocytes and lymphocytes to enter and leave the peripheral circulation [23]. Previous studies in adults measuring MMP-9 and TIMP-1 in sepsis have shown variable results. In two studies, plasma levels of both MMP-9 and TIMP-1 were significantly higher in patients with severe sepsis compared with healthy controls on day 1 of severe sepsis [6, 8]. Another study [7] showed that MMP-9 levels were significantly higher on admission and significantly reduced by day 3, and TIMP-1 was significantly elevated during the whole study period compared to controls. However, others have reported that MMP-9/TIMP-1 ratios were significantly lower at the first 3 days in 38 patients with severe sepsis compared to healthy controls [7] and that a lower MMP-9/TIMP-1 ratio was associated with greater sepsis illness severity and risk for mortality [22]. Our data suggest that this ratio is decreased in pediatric sepsis, can discriminate between septic children and healthy and febrile controls, and that a lower ratio is associated with higher morbidity and length of stay. While MMP-9 levels in this study were higher in febrile controls compared to healthy patients, this was not statistically significant. Previous studies have compared patients with sepsis to healthy controls, but not to febrile controls [6, 8, 22]. Similarly, the significance of the increase in mrProANP in the febrile group compared to healthy controls is not clear as the group size is small and there are little published data for comparison.

Circulating levels of A-FaBP, an intracellular lipid-binding protein, have been linked to severity of atherosclerosis, cardiovascular disease events, ischemic stroke, and sepsis [10]. Previous studies in adults with sepsis showed higher A-FaBP levels in non-survivors [24]. There are no data on A-FaBP use in pediatric septic patients. Our results show a significant increase in A- FaPB levels in septic patients compared to healthy patients, but this biomarker did not discriminate between healthy and febrile patients. This finding may be as a result of an under-powered sample. These data suggest it is premature to conclude there is no utility of A-FaPB as a biomarker in clinical practice in the evaluation of children with suspected sepsis. Indeed, the presence of low MMP-9/TIMP-1 ratios or high MrProANP levels were associated with higher organ dysfunction scores and longer ICU and hospital LOS and A-FaBP level was associated with PELOD, hospital and ICU length of stay. These findings are congruent with results from studies of adults with sepsis in which reduced MMP-9/TIMP-1 ratios were associated with higher organ dysfunction scores (SOFA) and mortality [22, 25]. Studies of sepsis in adults have also shown an association between MrProANP and A-FaBP levels and Acute Physiology and Chronic Health Evaluation II scores [24, 26].

mrProANP showed promise as a biomarker to identify children with sepsis, with a graded increase observed from healthy to febrile to septic patients in our study. ProANP regulates volume and pressure homeostasis in the circulatory system, modulates endothelium permeability, and participates in the innate and acquired immunological response [27, 28, 29]. In adults, mrProANP increases progressively with the severity of sepsis and is a predictor of mortality in adult patients with sepsis related to ventilator associated pneumonia [26]. Pro-ANP levels in adults with severe sepsis and septic shock may be associated with prognosis but do not discriminate between patients with sepsis and those with septic shock [9].

Neurologic complications of sepsis in children are not well defined. As a result the extent of neurologic morbidity resulting from sepsis in childhood is not known. A long-term follow-up study of children who survived septic shock found 44% of the survivors had cognitive score < 25% of aged-matched healthy chidren [30]. Children with menengieoencephalitis and septic illness had deficits in neuropsychologic performance and educational difficulties 3–6 months after hospital discharge [31]. The biomarkers we selected have each been proposed as markers for the detection of neurologic injury in clinical and pre-clinical studies in adults including meningitis [11], stroke [12–14], and sepsis [15]. Both mrProANP and A-FaBP have been proposed as diagnostic and prognostic biomarkers in adults with stroke [16–18] We found higher A-FaBP levels in the septic subjects with neurologic dysfunction, but only MMP-9/TIMP-1 ratio was associated with 6-month outcome.

Numberous diagnostic biomarkers have been investigated in pediatric and adult sepsis, including procalcitonin (PCT) and C-reactive protein (CRP),either to identify bacterial infection or differentiate septic patients from those with SIRS [32–34]. A metaanalysis showed a moderate ability of PCT to diagnose sepsis in adult patients (AUC for PCT 0.78 vs CRP 0.71) [34]. In a pediatric study, the AUC for PCT was 0.99 compared to 0.54 for CRP to differentiate patients with SIRS from those with sepsis [35]. Similarly, elevated PCT is a senstiive measure to differentiate bacterial from non-bacterial SIRS in children [33]. While our study represents data from a single center and a small cohort, the AUC for these measures to identify septic patients (A-FaBP, 0.76; MMP9/TIMP-1, 0.96; mrProANP, 0.99) are promising. Nevertheless, the lack of association in the sepsis cohort between these meausures and an established biomarker (CRP) is a limitation of the present study and would need to be investigated in future, prospective studies evaluating these biomarkers for the early detection of children with sepsis.

Many biomarkers have been investigated in adults and children to detect sepsis-related neurological injuries, including S-100 *β*, neuron-specific enolase (NSE), and the astrocyte protein, glial fibrillary acidic protein (GFAP) [36, 37]. In a prospective study of neurologic injury in children with septic shock, a combination of neurologic examinations and serial electroencephalography monitoring was used to identify neurologic injury [36]. In adults with sepsis and septic shock, both serum S-100β and GFAP were elevated compared to controls. Increase in NSE and S-100β levels were associated with the maximum sequential organ failure assessment scores (SOFA) and early mortality [37]. Serum S-100β may be a more sensitive biomarker than NSE for detection of sepsis associated encephalopathy [38].

The mortality in the sepsis cohort in this report (7%) is lower than that reported (24%) in the SPROUT point prevalence study of severe sepsis [2], and in a multi-center cohort study of children requiring intensive care in Australia and New Zealand in which 11% of the combined sepsis and septic shock groups died [39]. The sepsis cohort used in this study was part of study investigating nitric oxide signaling in sepsis [19]. For this reason, certain high-risk groups with sepsis were excluded (metabolic, urea cycle, or mitochondrial disorder, chronic renal or hepatic impairment, unrepaired cyanotic heart disease or single-ventricle anatomy, major surgery within the previous 72 hours, transfer from another facility with ongoing sepsis >24 hours). The exclusion of these patients may account for the lower mortality in our cohort.

This study has several limitations. Since residual specimens were used for analysis, samples were not available for all patients, requiring us to pool day 1 and 2 blood in some cases. We did not record the specific times within this 48 hour hour window when these samples were obtained. Thus, these results reflect a time window during which both the processes of sepsis and therapies are changing rapidly, and confound these results. Because of the small sample size in this pilot study, we were not able to test for an effect of confounding factors on boimarker results. The retrospective analysis of neurologic complications and the short term outcome limits any definitive conclusions about the association of these biomarkers with neurologic complications of sepsis.

## Conclusions

We found that MMP-9/TIMP-1 ratios were significantly lower, while A-FaBP and mrProANP levels were significantly higher in septic children compared to febrile and healthy controls. All three biomarkers were associated with morbidity, and length of stay. These results suggest that these biomarkers merit further prospective study for the early identification of children with sepsis.
